# Mapping Census Tract Clusters of Type 2 Diabetes in a Primary Care Population

**DOI:** 10.5888/pcd16.180502

**Published:** 2019-05-16

**Authors:** Marynia Kolak, Geethi Abraham, Mary R. Talen

**Affiliations:** 1 Center for Spatial Data Science, Chicago, Illinois; 2 McGaw Medical Center of Northwestern University, Chicago, Illinois; 3 Erie Family Health Center, Chicago, Illinois

**Figure:**
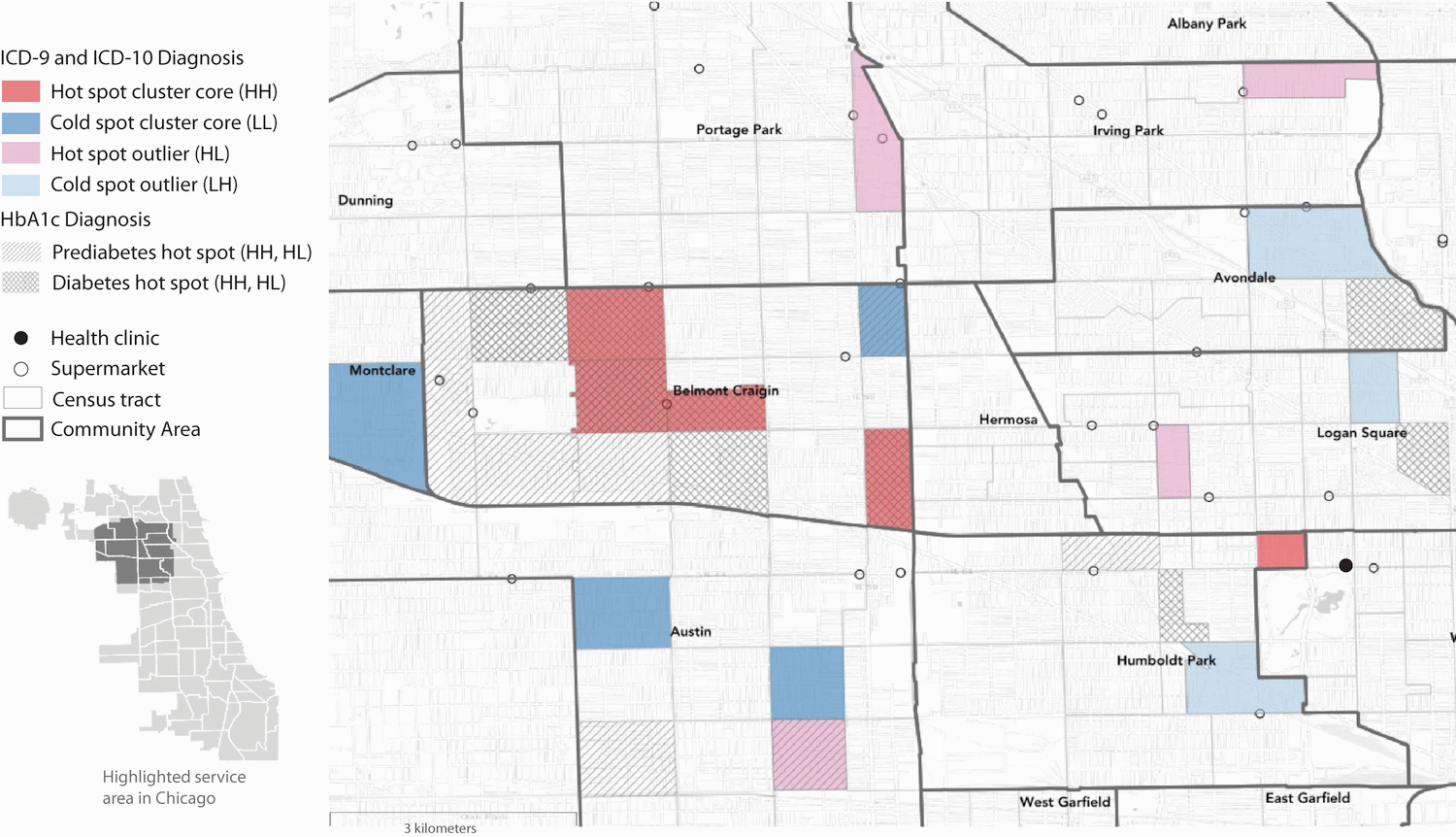
This map shows areas with significantly high and significantly low prevalence of diabetes and prediabetes in a health center population in Chicago. Prevalence was determined by ICD (*International Classification of Diseases*) codes and measured hemoglobin A1_c_ (HbA_1c_). The map highlights regional clusters and isolated areas of diabetes prevalence that could be targeted with interventions to improve health outcomes. Diagnoses determined by ICD codes are shown in colors as hot and cold spot cluster cores corresponding to “high–high” (HH) and “low–low” (LL) LISA (local indicator of spatial autocorrelation) statistics, where selected census tracts and neighboring tracts both have high rates (HH) or both have low rates (LL) of diabetes. Hot spot outliers have high diabetes rates compared with neighboring tracts (“high–low” [HL]), whereas cold spot outliers have low diabetes rates compared with neighboring tracts (“low–high” [LH]). Hot spots of prediabetes and diabetes determined by measured HbA_1c_ levels are also shown. LISA significance set at *P* < .05. Supermarket data are from Kolak et al (1). Census tract and community area boundary data are from the Chicago Data Portal (2,3). Basemap imagery is from OpenStreetMap, Leaflet, and Carto.

## Background

Although much effort has been made by public health agencies to geographically plot chronic diseases at the national, state, and city levels, information is limited on how disease is distributed in smaller geographic areas or populations, such as a health center population (4). Our objective was to identify patterns of type 2 diabetes in a patient population of a large urban federally qualified health center by using census tracts as a proxy for neighborhoods. This approach expands on other longitudinal studies that found associations between rates of type 2 diabetes and neighborhood social and physical environment characteristics such as access to healthy foods (5–7). 

As public health data have become more available, the spatial analyses of disease prevalence at local levels using clinical records has emerged as a powerful population-based health tool (8,9). Adapting these best methodological practices to an individual health center may provide additional strategies for identifying localized areas of health risk for targeting interventions and improving care in a health center’s population. Ultimately, merging GIS (geographic information systems) capabilities with primary care patient panels is a novel approach to guide disease management strategies and community outreach in a local health care system.

## Date Sources and Map Logistics

We generated maps from data extracted from the electronic medical records (N = 10,523) of a primary care patient population seen from August 1, 2015, to September 30, 2017, at a health center in Chicago. Residential addresses of patients were geocoded and converted to spatial data points. Points were then joined and aggregated to Chicago census tract boundaries (2). We created a subset of census tracts as the core service area; 31% of all patient-residing census tracts (140 of 455 tracts) included most of the health center’s patients (n = 9,126) and were located within 5 miles of the health center. This selection process reduced the number of spurious census tract outliers (ie, those with few health center patients). The study was approved by the Northwestern University Institutional Review Board.

We identified 1,246 patients with a type 2 diabetes diagnosis using the *International Classification of Diseases (ICD), Ninth Revision, Clinical Modification* codes 250.xx and *ICD, Tenth Revision*,* Clinical Modification* codes E11.XX; 854 of these patients resided in the core service area. We also classified patients by measured hemoglobin A1_c_ (HbA_1c_) levels; patients with levels from 5.4% to 6.4% were classified as having prediabetes, and patients with levels of 6.5% or higher were classified as having diabetes. Fifteen percent of patients in the core service area had HbA_1c_ levels ranging from 5.4% to more than 14%; in the wider population, 25% of patients were classified as such. On the basis of HbA_1c_ levels, 959 patients (of the total population) had type 2 diabetes, of whom 49 did not have an ICD code for diabetes. We used 5-year averages from the 2016 American Community Survey, as prepared by the Centers for Disease Control and Prevention’s Social Vulnerability Index database (10), for the following social determinants of health: percentage of persons living in poverty, rate of unemployment, per capita income, percentage of persons with no high school diploma, percentage of single parents, percentage limited in speaking English, percentage living in crowded housing, percentage having no vehicles, and percentage uninsured. Data for these covariates were extracted and joined to data for census tract areas.

We used exploratory spatial data analysis techniques on raw and population-adjusted data to analyze variation in type 2 diabetes distribution by census tract. We conducted an empirical Bayes smoothed univariate cluster and outlier detection analysis using the local indicator of spatial autocorrelation (LISA) statistic to determine type 2 diabetes prevalence (11). Empirical Bayes smoothing analysis uses a prior distribution, in this case, the average value of the sample, corrected for the variance instability associated with rates that have a small population base. We determined hot and cold spot clusters of type 2 diabetes prevalence. Hot spot clusters refer to areas that are significantly similar to their neighbors in high disease prevalence (“high–high” [HH] statistic), and cold spot clusters refer to areas that are significantly similar to their neighbors in low disease prevalence (“low–low” [LL] statistic). Clusters are composed of both cluster cores and nearby neighbors. Hot and cold spot clusters were visually identified by their cluster core in our analysis; these cores are surrounded by areas with proportionally high or low values. We also determined hot and cold spot outliers (“high–low” [HL] and “low–high” [LH]), which refer to areas that are significantly different (at *P* < .05) from their neighbors.

We compared the mean values of social determinants of health indicators in diabetes hot and cold spot tracts clusters with the mean values in all other core-service–area tracts using analysis of variance. We selected cluster cores and their neighboring tracts to represent complete clusters in this descriptive analysis. We added supermarket locations to the map as a proxy for access to healthy foods to demonstrate how incorporating environmental features may be useful in evaluating disease distribution (6). Although features other than supermarkets may be associated with disease occurrence, our analysis explored how clusters of type 2 diabetes may intersect with physical locations for food access. We used free and open-source software (R [The R Foundation] and GeoDa version 1.12.1.59 [Center for Spatial Data Science], a mapping and spatial statistics software) for all data management and analysis (12,13). We generated final maps by using R and Adobe Illustrator version 22.1.

## Highlights

Among 140 census tracts, we found 31 hot spot clusters and 25 cold spot clusters of patients with type 2 diabetes, along with 4 hot outliers and 3 cold outliers. Compared with all census tracts in the core service area, the census tracts in the hot spot clusters had significantly lower income levels and high school graduation rates and a significantly greater percentage of households with vehicles, single parents, residents with limited English proficiency, crowded housing, and persons without health insurance (Table). The raw type 2 diabetes rate in hot spot census tracts was significantly higher than in all other tracts (0.14 in 36 hot spots vs 0.10 in 104 non–hot spots; *P* = .005) and double the rate in cold spot census tracts (0.14 vs 0.07). We also found consistent overlap between hot spot census tracts and higher HbA_1c_ ranges; conversely, some tracts with high HbA_1c_ rates were not identified as hot spot tracts.

**Table:** Descriptive Statistics of Social Determinant of Health Covariates for Hot and Cold Spot Clusters of Diabetes in a Patient Population of a Large Health Center in Chicago, 2015–2017^a^

Determinant^b^	Hot Spot Cluster of Diabetes (n = 31)	Cold Spot Cluster of Diabetes (n = 25)	All Census Tracts in Primary Service Area (n = 140)
Poverty, %	22.5 (.64)	25.1 (.39)	23.3
Unemployment, %	13.4 (.73)	17.4 (<.001)	13.0
Per capita income, $	17,014 (.003)	16,727 (.006)	21,192
No high school diploma, %	34.7 (<.001)	28.3 (.12)	25.1
Single parent, %	16.4 (.16)	17.1 (.08)	14.6
Limited in speaking English, %	22.4 (<.001)	11.9 (.17)	14.3
Crowded housing, %	8.9 (<.001)	5.0 (.23)	6.1
No vehicles, %	18.1 (.02)	21.2 (.57)	22.4
Uninsured, %	26.6 (.005)	23.0 (.59)	23.7

a Diabetes data extracted from the electronic medical records (n = 10,523) of a primary care patient population seen from August 1, 2015, to September 30, 2017. *P* values, determined by analysis of variance, are for difference between means for the cluster (hot spot or cold spot) and means for all other tracts in the sample. An average for all tracts in the service area is provided for additional context.

b 5-year averages from the 2016 American Community Survey (10).

## Action

Our analysis demonstrates that stable calculation at the census tract level of a health center’s patient population can facilitate spatial analysis and identify patient groups with health risks in neighborhood clusters. This research can set a foundation for developing targeted interventions in a health center population at the neighborhood level to improve health outcomes. Our maps also identified neighborhoods at lower risk for type 2 diabetes and create opportunities to explore possible neighborhood resiliency factors.

Our data and findings represent a patient population and are not meant to serve as a true sample of the actual population. However, comparing the differences between hot and cold spot clusters can open avenues for mobilizing outreach at community health centers or federally qualified health centers and partnering among local resources to support interventions for the clinical population. Findings can be shared with policy makers and community advocates to influence what resources are needed and where they are needed most. Although geographically plotting of chronic diseases at the national, state, and city levels provides important information on the distribution of disease among populations, our map illustrates the use of exploratory data analysis techniques to expand the use of patient panels or registries and identify and address the health needs of vulnerable patient populations within a health system at the local level. This methodology can build bridges and partnerships between community health centers, federally qualified health centers, public health officials, and community organizations to develop neighborhood initiatives and outreach.

## References

[1] Kolak M , Bradley M , Block D , Pool L , Garg G , Toman CK , Chicago supermarket data and food access analytics in census tract shapefiles for 2007–2014. Data Brief 2018;21:2482–8. 10.1016/j.dib.2018.11.014 30560157 PMC6288981

[2] Chicago Data Portal. Boundaries — census tracts — 2010. https://data.cityofchicago.org/Facilities-Geographic-Boundaries/Boundaries-Census-Tracts-2010/5jrd-6zik. Accessed May 11, 2018.

[3] Chicago Data Portal. Boundaries — community areas (current). https://data.cityofchicago.org/Facilities-Geographic-Boundaries/Boundaries-Community-Areas-current-/cauq-8yn6. Accessed May 11, 2018.

[4] Wilkinson RG , Pickett KE . Income inequality and socioeconomic gradients in mortality. Am J Public Health 2008;98(4):699–704. 10.2105/AJPH.2007.109637 17901426 PMC2376999

[5] Hunt BR , Whitman S , Henry CA . Age-adjusted diabetes mortality rates vary in local communities in a metropolitan area: racial and spatial disparities and correlates. Diabetes Care 2014;37(5):1279–86. 10.2337/dc13-0988 24574350

[6] Christine PJ , Auchincloss AH , Bertoni AG , Carnethon MR , Sánchez BN , Moore K , Longitudinal associations between neighborhood physical and social environments and incident type 2 diabetes mellitus: the Multi-Ethnic Study of Atherosclerosis (MESA). JAMA Intern Med 2015;175(8):1311–20. 10.1001/jamainternmed.2015.2691 26121402 PMC4799846

[7] Gebreab SY , Hickson DA , Sims M , Wyatt SB , Davis SK , Correa A , Neighborhood social and physical environments and type 2 diabetes mellitus in African Americans: The Jackson Heart Study. Health Place 2017;43:128–37. 10.1016/j.healthplace.2016.12.001 28033588 PMC5774670

[8] Green C , Hoppa RD , Young TK , Blanchard JF . Geographic analysis of diabetes prevalence in an urban area. Soc Sci Med 2003;57(3):551–60. 10.1016/S0277-9536(02)00380-5 12791496

[9] Dijkstra A , Janssen F , De Bakker M , Bos J , Lub R , Van Wissen LJ , Using spatial analysis to predict health care use at the local level: a case study of type 2 diabetes medication use and its association with demographic change and socioeconomic status. PLoS One 2013;8(8):e72730. 10.1371/journal.pone.0072730 24023636 PMC3758350

[10] Centers for Disease Control and Prevention, Agency for Toxic Substances and Disease Registry, Geospatial Research, Analysis, and Services Program. Social vulnerability index 2016 database Illinois. https://svi.cdc.gov/data-and-tools-download.html. Accessed May 1, 2018.

[11] Anselin L . Local indicators of spatial association — LISA. Geogr Anal 1995;27(2):93–115. 10.1111/j.1538-4632.1995.tb00338.x

[12] Anselin L , Syabri I , Kho Y . GeoDa: an introduction to spatial data analysis. Geogr Anal 2006;38(1):5–22. 10.1111/j.0016-7363.2005.00671.x

[13] Ihaka R , Gentleman RR . a language for data analysis and graphics. J Comput Graph Stat 1996;5(3):299–314.

